# Nisin or Chitosan Enhance the Antimicrobial Activity of Ceftiofur Against Antibiotic-Resistant *Staphylococcus aureus* and Have Antibiofilm Effects

**DOI:** 10.3390/pathogens14121217

**Published:** 2025-11-29

**Authors:** Mónica G. Sánchez-Ceja, Jaime L. Esquivel-Alejo, Ricardo I. Medina-Estrada, Rafael Jiménez-Mejía, Gustavo Santoyo, Joel E. López-Meza, Pedro D. Loeza-Lara

**Affiliations:** 1 Foods Genomics, Universidad de La Ciénega del Estado de Michoacán de Ocampo (UCEMICH), Sahuayo 59103, Mexico; monnysanchezc@hotmail.com (M.G.S.-C.); jaimeluisesquivelalejo35@gmail.com (J.L.E.-A.); rimedina@ucemich.edu.mx (R.I.M.-E.); rjimenez@ucemich.edu.mx (R.J.-M.); 2 Genomic Diversity Laboratory, Institute of Chemical and Biological Research, Universidad Michoacana de San Nicolás de Hidalgo, Morelia 58030, Mexico; gustavo.santoyo@umich.mx; 3 Multidisciplinary Center for Biotechnology Studies, Universidad Michoacana de San Nicolás de Hidalgo, Morelia 58893, Mexico; elmeza@umich.mx

**Keywords:** *Staphylococcus aureus*, antibiotic resistance, ceftiofur, nisin, chitosan, antibiotic enhancers

## Abstract

Mastitis is one of the major diseases affecting dairy cattle worldwide. Antibiotic therapy remains the most widely used treatment. However, its effectiveness has been compromised due to the selection of antibiotic-resistant and biofilm-producing pathogenic bacteria. This promotes the search for alternatives that increase the antibacterial and antibiofilm efficacy of antibiotics such ceftiofur (CFT). Nisin (N) and chitosan (CH) may possess these properties. The aim of this study was to evaluate whether N + CFT and CH + CFT combinations enhance the antibacterial activity of the antibiotic on *Staphylococcus aureus* associated with bovine mastitis, as well as its antibiofilm effect. Two clinical isolates of *S. aureus* (AMC-43 and AMC-48) and the reference strain ATCC 27543 resistant to CFT were used. Through the microdilution method in 96-well microplates, the combination of sub-inhibitory concentrations of N (320 µg/mL) and CH (400 µg/mL) with CFT (1, 2, 4, and 8 µg/mL) significantly reduced bacterial growth; however, the CH + CFT mixtures were the most efficient. The crystal violet staining method and live cell plating showed antibiofilm activity in biofilm synthesis and in the reduction in living bacterial cells located inside this preformed structure. These results highlight N and CH as potential agents for the prevention or control of bovine mastitis.

## 1. Introduction

Mastitis or the inflammation of the mammary gland is the most costly and prevalent disease in dairy herds. Globally, millions of dollars are lost annually to the disease which markedly impacts dairy farming. The multifactorial etiology of the disease complicates its treatment. However, Gram-positive and Gram-negative microorganisms are prominent as causal agents [1]. Among these, the most important species capable of causing inflammation belong to a few groups, which are categorized as contagious and environmental pathogens according to their reservoir and transmission mechanisms [2].

*Staphylococcus aureus* is the most frequently isolated contagious pathogen from milk samples of cows with clinical and subclinical mastitis [3]. This pathogen causes the most virulent forms of the disease and poses one of the greatest challenges to milk production worldwide [4]. Antibiotic therapy is the primary strategy used to control bacterial infections, especially β-lactam antibiotics such as ceftiofur and oxacillin [5]. However, this therapy fails due to prolonged and inappropriate use of antibiotics. This increases bacterial selection pressure, which has led to the development of resistant strains. Added to this is the rapid spread of resistance determinants among pathogenic microorganisms [6].

Resistance of *S. aureus* strains to penicillin and its variants implicated in mastitis (β-lactams) is perhaps the most recognized form of resistance, ranging between 30 and 90% with variations from one region to another where dairy herds exist [7]. *S. aureus* strains such as methicillin-resistant *S. aureus* (MRSA) that are resistant to multiple antibiotics are common [6]. During bovine mastitis development, some *S. aureus* strains utilize virulence factors, such as biofilm matrix, to establish themselves in the host [8]. Furthermore, biofilms might provide protection against antimicrobial strategies owing to the low diffusion capacity of antibiotics through biofilm components [9]. Both bacterial strategies, resistance to antibiotics and biofilm synthesis, allow the development of recurrent intramammary infections. Lack of novel antibiotics from the pharmaceutical industry constitutes a major challenge for the veterinary sector and global public health [5].

All of this underlines the need to integrate innovative strategies to improve the efficacy of traditional antibiotics, inhibit biofilm synthesis, and help control recurrent intramammary infections. Biocompounds that, combined with antibiotics at sub-inhibitory concentrations, improve the antibiotic’s efficacy against resistant bacteria, possess antibiofilm properties, and are safe for human and animal health are receiving increasing attention [10,11]. Nisin is a cationic peptide of 34 amino acids produced by *Lactococcus lactis* subsp. *lactis*, with antibacterial activity, particularly against Gram-positive bacteria. Recognized as a safe additive, this peptide has been proposed as a potential therapeutic agent for control of bacterial infections of the mammary gland [12]. Chitosan is a cationic polymer of natural origin with biological activity against Gram-positive and Gram-negative pathogens [13]. This biodegradable and nontoxic biocompound has been proposed for various biomedical therapies [14]. Both biocompounds have exhibited antibacterial activity against bovine-mastitis-associated *S. aureus* isolates [7].

However, their ability to enhance the antibacterial effect of conventional antibiotics, like ceftiofur, and their effects on biofilm synthesis and the bacterial viability inside the biofilm remain unknown. Therefore, the aim of this study was to assess the enhancer effect of the combination of nisin or chitosan with ceftiofur on its antibiotic activity on antibiotic-resistant *S. aureus*, as well as the effect of the combinations on biofilm synthesis and bacterial viability within this preformed structure.

## 2. Materials and Methods

### 2.1. Bacterial Isolates and Growth Conditions

Two isolates of bovine-mastitis-associated *S. aureus* including AMC-43 and AMC-48 were used. These clinical isolates were obtained from milk samples from cows diagnosed with clinical and subclinical mastitis (California mastitis test) from 34 dairy herds in the Ciénega de Chapala region, Michoacán, Mexico. The isolates were characterized microscopically (morphology), biochemically (Gram staining, catalase, coagulase, tellurite reduction, and lipolytic activity tests in Petri dishes with Baird Parker agar and growth on salt and mannitol agar), and molecularly (amplification of a fragment of the *nuc* gene, which encodes the thermostable nuclease of *S. aureus*) [7]. The certified *S. aureus* subsp. *aureus* ATCC (27543) strain, which was isolated from a clinical case of bovine mastitis, was used as positive control. Bacteria were cultured in nutrient agar (BD Bioxon^®^, Ciudad de México, Mexico) at 37 °C for 24 h and maintained at 4 °C until further use. Due to the nature of the research and the way the milk samples were obtained, approval from an ethics committee was not required.

### 2.2. Nisin and Chitosan Antibacterial Activity and Ceftiofur Resistance Assays

Following the manufacturer’s instructions, the antimicrobial peptide nisin (N) (Nisina Raff^®^, Zapopan, Jalisco, Mexico) was dissolved in distilled water and sterilized by filtrating through 0.22 µm pore diameter membranes (Swinnex^®^, Darmstadt, Germany, Merck Millipore^®^). On the other hand, polymer chitosan (CH) (≥90% deacetylation grade, Future Foods^®^, Ciudad de México, Mexico) was dissolved in 1% acetic acid (J.T. Baker^®^, Radnor, PA, USA), continuously stirred at 500 rpm for 6–8 h until the mixture was completely homogeneous. The solution pH was adjusted to 5.6 with NaOH (J.T. Baker^®^, USA) and sterilized at 120 °C for 15 min. The β-lactam antibiotic ceftiofur (CFT) (Vetranal^®^, Sigma Aldrich, St. Louis, MO, USA) was dissolved in sterile distilled water. The biocompounds and antibiotic were kept at 4 °C until further use.

Minimum inhibitory concentration (MIC) and minimum bactericidal concentration (MBC) of N, CH, and CFT were determined using the microdilution method in Mueller–Hinton broth (MHB) (BD Bioxon^®^, Mexico) in 96-well microplates (Celltreat^®^, Ayer, MA, USA), following the Clinical Laboratory Standards Institute (CLSI) [15] guidelines and the methodology of Ster et al. [16] with some modifications. Overnight bacterial cultures in MHB at 37 °C were adjusted to ~0.2 optical density (OD) at 600 nm (Thermo Fisher Scientific^®^ spectrophotometer, Waltham, MA, USA), equivalent to approximately 4.8 × 10^6^ CFU/mL (6.68 log). These cultures were incubated for 24 h at 37 °C in 96-well microplates in different concentrations of N, CH, and CFT with a final volume of 150 µL per well. Concentration ranges of 10–5120, 12.5–6400, and 1–2048 µg/mL were used for N, CH, and CFT, respectively. The highest concentrations were serially diluted (doubling dilutions) [5,16,17]. Resistance to CFT was confirmed by inoculating bacteria on Mueller–Hinton agar (MHA) plates containing the indicated antibiotic concentrations.

MIC was defined as the minimum concentration of antibiotic or biocompounds that prevented bacterial growth: the concentration whose OD was equal to that inoculated (~0.2) after 24 h of incubation. Afterwards, the content of each well equal to or greater than the MIC was streaked on MHA plates and incubated at 37 °C to analyze bacterial growth 24 h after plating. The lowest concentration without visible bacterial colony growth was considered the MBC. *S. aureus* was considered resistant to CFT when it grew at a concentration ≥ of 8 µg/mL after 24 h of incubation [5,16,17]. MHB without *S. aureus* was used as a negative control of bacterial growth, while MHB containing *S. aureus* (~4.8 × 10^6^ CFU/mL) and MHB containing *S. aureus* (~4.8 × 10^6^ CFU/mL) and CFT (8 µg/mL) were used as a positive growth control. For CH, a second positive control of bacterial growth was used: MHB containing *S. aureus* (~4.8 × 10^6^ CFU/mL), and the vehicle of CH (1% acetic acid, pH 5.6). The treatments evaluated were MHB with *S. aureus* (~4.8 × 10^6^ CFU/mL) and the following N concentrations: 5120, 2560, 1280, 640, 320, 160, 80, 40, 20, and 10 µg/mL; as well as MHB with *S. aureus* (~4.8 × 10^6^ CFU/mL) and the following CH concentrations: 6400, 3200, 1600, 800, 400, 200, 100, 50, 25, and 12.5 µg/mL; and MHB with *S. aureus* (~4.8 × 10^6^ CFU/mL), as well as the next concentrations of the antibiotic CFT: 2048, 1024, 512, 256, 128, 64, 32, 16, 8, 4, 2, and 1 µg/mL.

### 2.3. Inhibition Assays of S. aureus by N, CH, CFT, and N + CFT and CH + CFT Combinations

The antibacterial effect of N + CFT and CH + CFT combinations on antibiotic-resistant *S. aureus* was investigated using the microdilution method. Overnight bacterial cultures in MHB at 37 °C were adjusted to an OD of ~0.2 and incubated for 24 h at 37 °C in the presence of sub-inhibitory concentrations (sub-MICs) of N (320 µg/mL) and CH (400 µg/mL), combined with different concentrations of CFT (1, 2, 4, and 8 µg/mL), which represent the CFT breakpoint for *S. aureus* (8 μg/mL), while the rest are lower than this breakpoint (1, 2, and 4 μg/mL). For example, (320 + 1) means that the combination contained 320 µg/mL of N and 1 µg/mL of CFT, and so on. This also applied to the mixture of CH with CFT (400 + 1). The positive and negative controls for bacterial growth were MHB + *S. aureus* and MHB − *S. aureus*, respectively. The following treatments were evaluated: MHB + *S. aureus* + N; MHB + *S. aureus* + CH; MHB + *S. aureus* + N + CFT; MHB + *S. aureus* + CH + CFT. The reduction in bacterial growth was verified by measuring the OD of each treatment and quantifying the CFU/mL, which were expressed in logarithmic units, after 24 h of incubation in MHA plates. In addition, the logarithmic scale reduction factor (Log_10_) was calculated using Formula (1):

(1) $$RF={Log}_{10}\left(A\right)-{Log}_{10}(B)$$

where *A* is the number of colonies recovered from the unexposed (control) and *B* is the number of colonies recovered from the exposed (test) to treatments. Using the average values of these counts, the microbial reduction percentages (MRP) were calculated according to Formula (2) [18]:

(2) $$MRP=\frac{\left(control\frac{CFU}{mL}-test\frac{CFU}{mL}\right)}{control\frac{CFU}{mL}}\times 100$$

### 2.4. Biofilm Production Assays of S. aureus and Inhibition by N, CH, CFT, and N + CFT and CH + CFT Combinations

To assess the inhibitory capacity of N, CH, CFT, and their corresponding combinations on the synthesis of *S. aureus* biofilms, the ability of the bacteria to produce this virulence factor was determined, followed by inhibition analysis. Biofilm synthesis was determined using the crystal violet staining methodology reported by Guzmán-Rodríguez et al. [19], with some modifications. Assays were performed in 96-well plates, where 150 µL of each bacterial culture grown in MHB adjusted to an OD of ~0.2 (600 nm) was placed. The plates were incubated for 24 h at 37 °C. Next, the wells were washed with phosphate-buffered saline (PBS), 150 µL of 0.5% crystal violet (Golden Bell^®^, Guadalajara, Mexico) was added, and plates were maintained for 15 min at room temperature (25 ± 2.0 °C). Subsequently, the dye was discarded, and the wells were washed twice with PBS. Finally, 150 µL of 33% acetic acid (Merk^®^, Naucalpan de Juárez, Estado de México, Mexico) was added, the plate was thoroughly mixed for homogenization, and the absorbance was read at 595 nm using a microplate spectrophotometer. The classification by Stepanović et al. [20] was used to categorize bacteria as non-biofilm producers (0), weak biofilm producers (+ or 1), moderate biofilm producers (++ or 2), and strong biofilm producers (+++ or 3) based on the OD values of the samples analyzed according to the following calculations:

OD≤ODcontrol=non−biofilm producer;

ODcontrol<OD ≤2×ODcontrol=weak biofilm producer;

2×ODcontrol<OD ≤4×ODcontrol=moderate biofilm producer;

4×ODcontrol<OD=strong biofilm producer

where *ODcontrol* is the OD of the broth without bacteria.

Then, the antibiofilm effects of the biocompounds, antibiotic, and their combinations were determined using the same methodology of Guzmán-Rodríguez et al. [19], except that, in this case, bacterial cultures grown in MHB and adjusted to an OD of ~0.2 were added to the 96-well plates, along with sub-MIC concentrations of N (320 µg/mL), CH (400 µg/mL), and the breakpoint concentration of CFT (8 µg/mL), as well as the corresponding N + CFT, and CH + CFT combinations. The plates were incubated for 24 h at 37 °C. The positive and negative controls for biofilm formation were MHB + *S. aureus* and MHB − *S. aureus,* respectively. The treatments included MHB + *S. aureus* + N; MHB + *S. aureus* + CH; MHB + *S. aureus* + N + CFT; and MHB + *S. aureus* + CH + CFT. The antibiofilm effects of the treatments were calculated according to Formula (3):

(3) $$Inhibition\;\left(\%\right)=1-\left(\frac{ODtreatment}{ODcontrol}\right)\times 100$$

where *ODcontrol* represents the maximum biofilm production of each isolate at the time of spectrophotometric reading. Likewise, the effect of the combinations on this virulence factor allowed the bacteria to be recategorized according to Stepanović et al. [20].

### 2.5. Antibacterial Activity on S. aureus Cells Living Within Preformed Biofilms

These experiments were only performed on the clinical isolate of *S. aureus* that was classified as the strongest biofilm producer (AMC-48). Bacterial cultures grown in MHB and adjusted to an OD of ~0.2 were added to the 96-well plates. Plates were statically incubated for 24 h at 37 °C to allow cell adhesion and biofilm synthesis. After the incubation period, supernatants were removed and 150 μL of sterile PBS was added (twice) to remove non-adherent bacteria. The preformed biofilms were treated with N, CH, CFT, and their corresponding combinations for 24 h at 37 °C. Once again, the supernatant was removed, and the biofilm was washed with 150 μL of sterile PBS (twice). After these washes, 150 μL of 0.1% Triton X-100 (Hycel^®^, Zapopan, Jalisco, Mexico) was added to break down the extracellular matrix and disperse the biofilm produced. The microplate was sealed with parafilm and placed in a sonicator (Branson Ultrasonics CPX-952-238R^®^, Brookfield, CT, USA) to 42 kHz by 5 min. Dilutions were then made and plated on MHA, incubated for 24 h at 37 °C, and the bacterial count was performed, which was expressed as logarithmic units [21].

### 2.6. Statistical Analysis

A completely randomized experimental design was used, comprising three independent experiments (biological replicas) with three repeats per treatment. Data were analyzed using analysis of variance, with a significance level of (*p* ≤ 0.05), followed by Tukey’s test (*p* ≤ 0.05). The percentages of biofilm synthesis inhibition were transformed using square root transformation (√ × + 0.5). Statistical analyses were performed using IBM SPSS Statistics version 25.

## 3. Results

### 3.1. Resistance and Susceptibility of S. aureus to CFT, N, and CH

The three bacterial members of *S. aureus* exhibited resistance to CFT, since ATCC 27543, AMC-43, and AMC-48 grew in the presence of ≥8 µg/mL of the antibiotic after 24 h of incubation (Table 1). CFT MICs ranged from 256 to 2048 µg/mL, while MBCs ranged from 512 to 2048 µg/mL. On the other hand, N exhibited antibacterial effect only against the ATCC 27543 strain, with an MIC of 640 µg/mL; however, the MBC at the highest peptide concentration used (5120 µg/mL) could not be determined. MICs and MBCs could not be determined for AMC-43 and AMC-48 either. In contrast, CH showed antibacterial effects against all evaluated bacteria, whose MICs and MBCs were 6400 µg/mL (Table 1).

### 3.2. Increasing the Efficacy of CFT Against S. aureus by the Addition of N and CH

The use of biocompounds as enhancers of the antibacterial effect of antibiotics is based on the use of sub-MICs of the former. To test the hypothesis that sub-MIC concentrations of N and CH increase the efficacy of CFT, the next concentrations were selected because the antibacterial effect on *S. aureus* was minimal or not significant: N (320 µg/mL) and CH (400 µg/mL). These were combined with CFT (1, 2, 4, and 8 µg/mL), and their effects on bacterial growth, biofilm synthesis, and bacterial viability within biofilms were evaluated. Regarding the N and CFT mix, the results showed that all compounds’ combinations reduced bacterial growth (expressed as logarithmic reduction scale, LRS) of ATCC 27543, AMC-43, and AMC-48 compared to controls and individual treatments with N and CFT added individually (*p* ≤ 0.05) (Table 2). As can be seen, in strain ATCC 27543, the combinations of N + CFT showed logarithmic reductions in a range from 2.24 to 2.76 units. In the case of the AMC-43 clinical isolate, the reduction in bacterial growth was more pronounced, with decreases within the range of 4.35 to 4.86 units. A similar result was observed with the isolate AMC-48, whose reductions in bacterial growth were also substantial, with a range of 3.66 at 4.22 units (Table 2). These results indicate that N addition improved the efficacy of CFT by reducing the bacterial growth of the three pathogenic microorganisms in an inhibitory range of 30.9–66.7%, with a clear tendency towards bacterial reduction in all combinations.

In addition, mixtures of CH and CFT showed that all combinations also reduced bacterial growth of ATCC, AMC-43, and AMC-48 when compared to controls and individual CH and CFT treatments (*p* ≤ 0.05) (Table 3). As can be seen, in strain ATCC 27543, the CH + CFT combinations showed logarithmic reductions in bacterial growth of 7.21 units (0 CFUs recovered) in all combinations, showing substantial differences from the control, CH, and CFT alone. An identical result was observed with the clinical isolate AMC-43, since bacterial growth was also reduced to 7.39 units (0 CFUs recovered) in all combinations evaluated. Finally, although the inhibition of the AMC-48 isolate was less pronounced, this decrease was considerable, with a range of 4.96 to 5.67 units (Table 3). These results indicate that CH addition improved the efficacy of CFT by reducing the bacterial growth of the three bacterial pathogens in an inhibitory range of 66.7 to 100%, again, showing a clear trend towards bacterial reduction in all combinations. Importantly, the vehicle did not inhibit bacterial growth of any of the three pathogens, as shown in Table 3.

### 3.3. Inhibition of S. aureus Biofilm Synthesis After Adding N, CH, N + CFT, and CH + CFT

**Figure 1:**
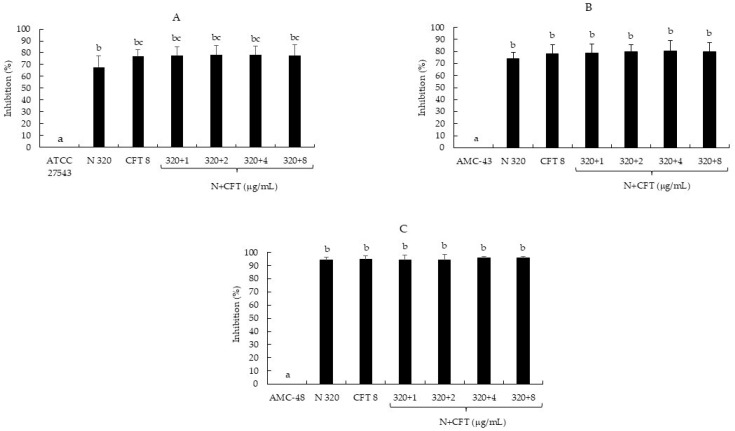
Inhibition percentage of *S. aureus* biofilm production by nisin (N), ceftiofur (CFT), and N + CFT after 48 h of incubation. (**A**) *S. aureus* ATCC 27543. (**B**) *S. aureus* AMC-43. (**C**) *S. aureus* AMC-48. Evaluation of different combinations of N + CFT are shown. Graph represents the mean ± standard deviation. Different letters within each column indicate significant differences according to Tukey’s test (*p* ≤ 0.05).

According to the classification of Stepanović et al. [20], the strain ATCC 27543 and isolate AMC-43 were classified as moderate biofilm producers (++), whereas isolate AMC-48 was considered a strong biofilm producer (+++) (Table 4). Regarding the effect of N, CFT, and N + CFT combinations on biofilm synthesis, all treatments inhibited the synthesis of this virulence factor in the three pathogenic bacteria evaluated, compared to the untreated controls (*p* ≤ 0.05) (Figure 1A–C). The inhibition percentage ranged from 67.8 to 95.9% among the three bacteria. However, no differences were observed in the inhibition of most combinations of N + CFT and individual treatments of N and CFT. These results show that it was not possible to detect an enhancing effect of N on CFT inhibiting biofilm synthesis. Nevertheless, one noteworthy aspect is that all treatments inhibited the synthesis of biofilm, which was additionally reflected in the recategorization of the bacteria as follows: *S. aureus* ATCC 27543 was reclassified from a moderate (OD = 0.29) to a weak producer (OD = 0.06); AMC-43 from a moderate (OD = 0.25) to a non-producer (OD = 0.05); and AMC-48 from a strong (OD = 1.28) to a weak producer (OD = 0.06) (Table 4).

**Figure 2:**
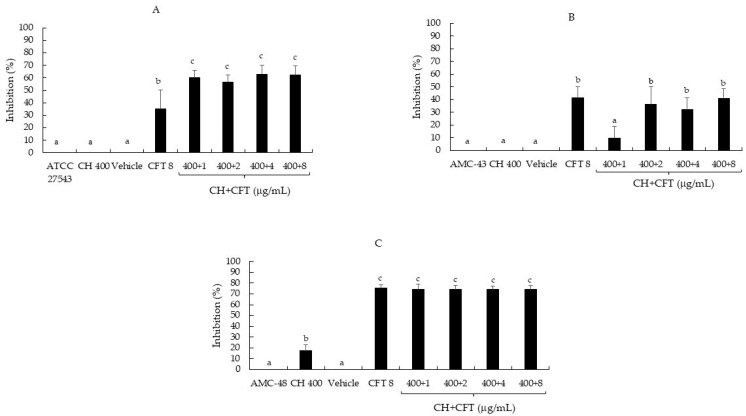
Inhibition percentage of *S. aureus* biofilm production by chitosan (CH), ceftiofur (CFT), and CH + CFT after 24 h of incubation. (**A**) *S. aureus* ATCC 27543. (**B**) *S. aureus* AMC-43. (**C**) *S. aureus* AMC-48. The vehicle used was: 1% acetic acid, pH 5.6. Evaluation of different combinations of CH + CFT are shown. Graph represents the mean ± standard deviation. Different letters within each column indicate significant differences according to Tukey’s test (*p* ≤ 0.05).

On the other hand, most CH + CFT combinations (91.6%) reduced biofilm synthesis compared to the untreated controls (*p* ≤ 0.05) (Figure 2A–C). The inhibition range on the three bacteria was 32.4 to 74.4%. When comparing the effect of the mixtures with the individual compounds, the trend showed differences between CH + CFT and CH alone, as inhibition was more pronounced in the combinations than in CH. However, this was not observed between CH + CFT and CFT alone, as the antibiotic inhibited biofilm synthesis (Figure 2A–C). Once again, these results show that it was not possible to detect an enhancing effect of CH on CFT in inhibiting the synthesis of *S. aureus* biofilm. Nevertheless, it is important highlight that the treatments that inhibited the synthesis of biofilm caused a recategorization of the bacteria as follows: *S. aureus* ATCC 27543 and AMC-43 were reclassified from moderate (OD = 0.29 and 0.25, respectively) to weak producers (OD = 0.11 and 0.18, respectively); and AMC-48 from a strong (OD = 1.28) to a moderate producer (OD = 0.34) (Table 4). Finally, it was also noted that the control (vehicle: 1% acetic acid, pH 5.6) showed no significant inhibition (*p* ≤ 0.05) of biofilm production in any of the microorganisms evaluated (Figure 2A–C).

**Figure 3:**
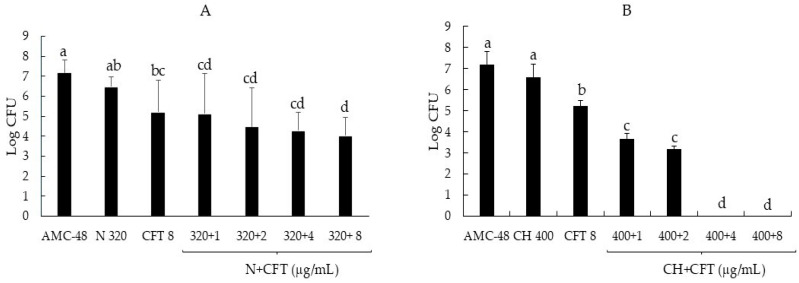
Reduction in the logarithmic growth of the AMC-48 isolate of *S. aureus* from inside the biofilm following the addition of biocompounds, antibiotic, and their combinations. (**A**) Nisin (N), ceftiofur (CFT), and their mixes. (**B**) Chitosan (CH), ceftiofur (CFT), and their mixes. Graph represents the mean ± standard deviation. Different letters indicate significant differences according to Tukey’s test (*p* ≥ 0.05).

In addition to analyzing the effect of combinations of biocompounds and CFT on the synthesis of biofilm produced by *S. aureus*, we were interested in determining whether these treatments could penetrate the preformed biofilm and reduce the CFUs inside the biofilm. The results showed that all N + CFT combinations reduced bacterial growth by up to 2.5 logarithmic units, showing significant differences compared to the control (*p ≤* 0.05) (Figure 3A). However, as shown in Figure 3B, the antibacterial effect of the CH + CFT combinations was more effective, as the combinations of 400 + 4 and 400 + 8, respectively, reduced the recovered CFUs to 0.

## 4. Discussion

Bovine mastitis caused by *S. aureus* affects dairy cattle worldwide and causes major economic losses [22]. As the disease is difficult to eradicate due, in part, to the high rate of bacterial resistance to antibiotic therapy and biofilm formation, studies should focus on the development of new antibiotics and sustainable management models. This can enhance antibiotic efficacy, reduce the evolution of natural resistance, and inhibit virulence factors, such as biofilm synthesis [23]. In this regard, molecules of natural origin, such as essential oils, antimicrobial peptides, carbohydrate polymers, and synthetic chemical compounds, are garnering attention as enhancers of antibiotic efficacy, inhibitors of antibiotic resistance, and virulence factors [5,12,24,25].

The present study contributes to this alternative by selecting two clinical isolates of *S. aureus* (AMC-43 and AMC-48) including the reference strain *S. aureus* ATCC 27543, because of their resistance to the β-lactam antibiotic CFT, as shown in Table 1. The MICs of CFT ranged from 256 to 2048 µg/mL, whereas the MBCs fluctuated between 512 and 2048 µg/mL. These results confirmed that the bacteria analyzed in this study were resistant to CFT, as the breakpoint for this antibiotic was 8 µg/mL [15,16]. This antibiotic is a third-generation cephalosporin that has been widely used for over two decades in mastitis treatment [26]. However, Molineri et al. [27] and Morar et al. [28] reported that increased bacterial resistance to conventional antibiotics over time has become a common characteristic, including resistance to CFT, which is consistent with the findings of this study.

Employing enhancers of the antibacterial effect of antibiotics relies on MIC or sub-MIC utilization. To select the sub-MIC of N and CH, the MICs of both biocompounds were also determined. The results showed an antibacterial effect of N only on the certified strain of *S. aureus* ATCC 27543, with an MIC of 640 µg/mL. Previous studies have reported the antibacterial effect of this peptide on bovine-mastitis-associated *S. aureus* [29]. The antimicrobial effect of N is based on its net positive charge, which allows it to bind to the peptidoglycan of the wall through electrostatic attractions and destabilizes it. This allows the peptide to insert into the bacterial cell membrane to form pores that cause the microorganism death [30]. Notably, the low susceptibility of clinical isolates to N is striking, since this peptide is not used in herds of the region as a therapeutic alternative against bovine mastitis. However, *S. aureus* has a high potential to develop natural resistance or low susceptibility to N in vitro [31]. This could explain its null antibacterial effect on the AMC-43 and AMC-48 isolates. However, further experimental work is needed to understand the mechanism of low susceptibility of the clinical isolates to N used in this study.

In contrast, CH showed antibacterial effects against all evaluated bacteria, whose MICs and MBCs were 6400 µg/mL (Table 1). CH is an efficient antibacterial agent that inhibits the growth of Gram-positive and Gram-negative bacteria [32]. These precedents include the antibacterial effect on bovine-mastitis-associated *S. aureus* [22], which corroborates the results of this study. The antibacterial activity of CH is based on its net positive charge, which allows it to efficiently bind to negatively charged cell wall components such as teichoic and lipoteichoic acids. This allows the formation of membrane pores and bacterial death [32].

Regarding the use of biocompounds as enhancers of the antibacterial effect of antibiotics, the results showed that all combinations of N + CFT reduced the bacterial growth (expressed as logarithmic reduction) of ATCC 27543, AMC-43, and AMC-48 compared to controls and individual treatments with N and CFT added individually (*p* ≤ 0.05) (Table 2). The increased efficacy of β-lactam antibiotics such as penicillin and CFT, in combination with N, has been evaluated against *S. aureus* (SA113), *S. pseudintermedius* (DSM21284), and *Streptococcus suis*, with positive results showing a greater reduction in bacterial growth [33,34]. However, this is the first study that evaluates the restoration of CFT’s antibiotic efficacy when combined with N on *S. aureus* associated with bovine mastitis. Notably, the combined use of antimicrobial agents with conventional antibiotics, which have different cellular targets, could reduce the selection of resistant bacteria, thereby potentially allowing for a reduction in antibiotic therapeutic doses [33]. In agreement with this statement, it is important to note that in this study no statistical differences were observed in the logarithmic reduction in bacteria between the combinations evaluated for each bacterium, suggesting that the combination with the lowest concentrations of CFT could be used, for example: 320 + 1 for N + CFT. However, further experiments are needed to determine the effectiveness of this combination. Furthermore, more experiments and analyses are needed to understand the type of interaction that allows this biological effect between the peptide and the antibiotic.

In addition, the CH and CFT mixtures showed that all combinations also reduced the bacterial growth of ATCC, AMC-43, and AMC-48 more efficiently compared to the controls and individual treatments with CH and CFT (Table 3). According to these results, other authors have reported an increase in the efficacy of antibiotics such as cloxacillin, oxacillin, ampicillin, penicillin, and cefotaxime when combined with CH or CH nanoparticles against coagulase-negative *Staphylococcus*, *Staphylococcus* spp., and enterobacteria [5,35,36]. However, this is the first study to evaluate the restoration of susceptibility of *S. aureus* associated with bovine mastitis to CFT, through the addition of subMIC CH. Similarly, these data suggest that combination therapy with CH and CFT could increase bacterial susceptibility, as well as the therapeutic dose of the antibiotic. In accordance with this assertion, it is important to note that in this study no statistical differences were observed in the logarithmic reduction in bacteria between the combinations evaluated for each bacterium, suggesting that the combination with the lowest concentrations of CFT could be used, for example: 400 + 1 for CH + CFT. Nevertheless, further experiments are needed to determine the effectiveness of this combination. Additionally, to provide further evidence to strengthen these results, it is necessary to know the type of interaction between CH and CFT, so additional analyses and studies are needed.

Regarding bacterial reduction expressed in logarithmic units, authors such as Boddie et al. [37], Fitzpatrick et al. [38], and the National Mastitis Council [39] indicate that a reduction of 3 units is acceptable, but a reduction of 4–5 logarithmic units is preferable. In this study, the reduction in the N + CFT combination ranged from 2.24 to 4.86 on the three bacteria. In this case, the combination would only be acceptable in the AMC-43 (4.35–4.86) and AMC-48 (3.66–4.22) clinical isolates, but not in the ATCC 27545 strain (2.24–2.76), so this combination should be evaluated in a larger number of clinical isolates of *S. aureus* to obtain more consistent data. On the other hand, the reduction in the CH + CFT combination ranged from 4.96 to 7.21 on all pathogenic bacteria. As can be seen, the combination is acceptable for ATCC 27543 (7.21), AMC-43 (7.39), and AMC-48 (4.96–5.67). Nevertheless, this combination should also be evaluated in a larger number of clinical isolates of *S. aureus* to strengthen the results of the CH + CFT combination.

Regarding the effect of the N, CFT, and N + CFT combinations on biofilm synthesis, all treatments inhibited the synthesis of this virulence factor in the three pathogenic bacteria evaluated compared to the untreated controls (*p* ≤ 0.05) (Figure 1A–C). The bacteria were even reclassified according to Stepanović et al. [20]’s categorization (Table 4). Similar to these results, another author has reported the effect of N sub-MICs in inhibiting biofilm synthesis by *S. aureus*, suggesting that this allows the peptide to inhibit biofilm synthesis by this pathogen without the need for a combination [40]. Additionally, Ster et al. [16] reported that concentrations below the CFT breakpoint (0.25 and 1.0 µg/mL) inhibit biofilm synthesis by *S. aureus*, which aligns with the findings in this study. On the other hand, most of the CH + CFT combinations (91.6%) reduced biofilm synthesis compared to the untreated controls (*p* ≤ 0.05) (Figure 2A–C). Once again, the bacteria were also reclassified (Table 4). The effect of CH on biofilm synthesis by *S. aureus* has been documented [41]. However, this is in contrast with our results, at least for ATCC 27543 and AMC-43, where no inhibition was observed. Nevertheless, CH concentrations that inhibit biofilm synthesis can reach up to 1600 µg/mL, which could explain the differences observed in this study, where only 400 µg/mL was used for CH [22].

Interestingly, when analyzing the effect of the combinations on the cells inside the biofilm, all N + CFT combinations reduced bacterial growth by up to 2.5 logarithmic units (Figure 3A). However, the antibacterial effect of the CH + CFT combinations was more effective, as the combinations of 400 + 4 and 400 + 8, respectively, reduced the recovered CFUs to 0 (Figure 3B). The above shows an enhancing effect of biocompounds on the antibacterial effect of CFT and suggests that these combinations appear to be penetrating the biofilm, as significant reductions in recovered CFUs were recorded. However, further studies are needed to strengthen this assumption. The results obtained in this study open new avenues for research. One of these is the evaluation of N + CFT and CH + CFT combinations in vivo, i.e., in dairy cattle, which could be evaluated as physical sealants for the mammary gland or as intramammary infusions.

## 5. Conclusions

This study demonstrated a significant increase in the antibacterial activity of the antibiotic CFT when combined with most of the sub-MICs of N and CH on the ATCC 27543 strain of *S. aureus* and the clinical isolates AMC-43 and AMC-48, all resistant to CFT and associated with bovine mastitis, which was reflected in the reduction in bacterial logarithmic units as recommended by the NMC. Although the results varied with the evaluated bacteria and the individual biocompounds, a reduction in biofilm synthesis by the three bacteria was observed with the N + CFT and CH + CFT combinations, as well as with the individual treatments of N, CFT, and to a lesser extent CH. However, it was not possible to detect a clear enhancing effect of the N + CFT and CH + CFT mixtures in inhibiting the synthesis of the *S. aureus* biofilm, in relation to the individual biocompounds—an effect that was well established in the cells living inside the biofilms. Finally, these findings indicate that N and CH could have potential applications in the reuse of CFT in dairy herds and could have possible uses in prevention and control (by using them as intramammary infusions or physical sealants of the mammary gland) of bovine mastitis caused by *S. aureus*.

## Figures and Tables

**Table 1:** Minimum inhibitory concentration (MIC) and minimum bactericidal (MBC) of ceftiofur (CFT), nisin (N), and chitosan (CH) against bovine-mastitis-associated *S. aureus*.

	CFT (μg/mL)		N (μg/mL)		CH (μg/mL)	
	MIC	MBC	MIC	MBC	MIC	MBC
ATCC 27543	256	512	640	ND ^a^	6400	6400
AMC-43	2048	2048	ND ^a^	ND ^a^	6400	6400
AMC-48	512	2048	ND ^a^	ND ^a^	6400	6400

^a^ ND: Not determined at the highest concentration evaluated in this study (5120 μg/mL). Values represent the means of three replicates and three independent experiments.

**Table 2:** Logarithmic growth reduction in *S. aureus* by nisin (N), ceftiofur (CFT), and N + CFT combination after 24 h of incubation.

	**ATCC 27543**						
Time (h) /tmts ^1^	Control ^2^	N 320	CFT 8	N + CFT 320 + 1	N + CFT 320 + 2	N + CFT 320 + 4	N + CFT 320 + 8
0	6.63 ± 0.08	6.63 ± 0.08	6.63 ± 0.08	6.63 ± 0.08	6.63 ± 0.08	6.63 ± 0.08	6.63 ± 0.08
24	7.26 ± 0.05 ^a^	7.34 ± 0.02 ^a^	6.31 ± 0.25 ^b^	5.02 ± 0.82 ^c^	4.50 ± 0.45 ^c^	4.55 ± 0.36 ^c^	4.65 ± 0.53 ^c^
RF ^5^	--	--	0.95	2.24	2.76	2.71	2.61
	**AMC-43**						
Time (h) /tmts ^1^	Control ^3^	N 320	CFT 8	N + CFT 320 + 1	N + CFT 320 + 2	N + CFT 320 + 4	N + CFT 320 + 8
0	6.56 ± 0.27	6.56 ± 0.27	6.56 ± 0.27	6.56 ± 0.27	6.56 ± 0.27	6.56 ± 0.27	6.56 ± 0.27
24	7.29 ± 0.26 ^a^	6.53 ± 0.09 ^ab^	5.08 ± 0.29 ^b^	2.94 ± 0.55 ^c^	2.43 ± 0.29 ^c^	2.72 ± 0.57 ^c^	2.54 ± 0.38 ^c^
RF ^5^	--	0.76	2.21	4.35	4.86	4.57	4.75
	**AMC-48**						
Time (h) /tmts ^1^	Control ^4^	N 320	CFT 8	N + CFT 320 + 1	N + CFT 320 + 2	N + CFT 320 + 4	N + CFT 320 + 8
0	6.81 ± 0.02	6.81 ± 0.02	6.81 ± 0.02	6.81 ± 0.02	6.81 ± 0.02	6.81 ± 0.02	6.81 ± 0.02
24	7.25 ± 0.09 ^a^	7.19 ± 0.05 ^a^	6.13 ± 0.23 ^b^	3.03 ± 0.76 ^c^	3.59 ± 1.05 ^c^	3.19 ± 0.86 ^c^	3.38 ± 0.97 ^c^
RF ^5^	--	0.06	1.12	4.22	3.66	4.06	3.87

Different letters indicate significant differences according to Tukey’s test (*p* ≥ 0.05). An analysis of variance (*p* ≥ 0.05) was performed for each bacterium. The data are presented as Log_10_ CFU/mL ± SD. ^1^ Treatments: µg/mL. ^2^ Control: *S. aureus* ATCC 27543 without treatment. ^3^ Control: *S. aureus* AMC-43 without treatment. ^4^ Control: *S. aureus* AMC-48 without treatment. ^5^ RF: Reduction factor.

**Table 3:** Logarithmic growth reduction in *S. aureus* by chitosan (CH), ceftiofur (CFT), and CH + CFT combination after 24 h of incubation.

**ATCC 27543**								
Time (h) /tmts ^1^	Control ^2^	Vehicle ^5^	CH 400	CFT 8	CH + CFT 400 + 1	CH + CFT 400 + 2	CH + CFT 400 + 4	CH + CFT 400 + 8
0	6.68 ± 0.30	6.68 ± 0.30	6.68 ± 0.30	6.68 ± 0.30	6.68 ± 0.30	6.68 ± 0.30	6.68 ± 0.30	6.68 ± 0.30
24	7.21 ± 0.06 ^a^	7.24 ± 0.07 ^a^	6.56 ± 0.02 ^b^	6.32 ± 0.26 ^c^	0 ± 0 ^d^	0 ± 0 ^d^	0 ± 0 ^d^	0 ± 0 ^d^
RF ^6^	--	--	0.65	0.89	7.21	7.21	7.21	7.21
**AMC-43**								
Time (h) /tmts ^1^	Control ^3^	Vehicle ^5^	CH 400	CFT 8	CH + CFT 400 + 1	CH + CFT 400 + 2	CH + CFT 400 + 4	CH + CFT 400 + 8
0	6.88 ± 0.07	6.88 ± 0.07	6.88 ± 0.07	6.88 ± 0.07	6.88 ± 0.07	6.88 ± 0.07	6.88 ± 0.07	6.88 ± 0.07
24	7.39 ± 0.16 ^a^	7.41 ± 0.16 ^a^	6.52 ± 0.34 ^b^	5.08 ± 0.29 ^c^	0 ± 0 ^d^	0 ± 0 ^d^	0 ± 0 ^d^	0 ± 0 ^d^
RF ^6^	--	--	0.87	2.31	7.39	7.39	7.39	7.39
**AMC-48**								
Time (h) /tmts ^1^	Control ^4^	Vehicle ^5^	CH 400	CFT 8	CH + CFT 400 + 1	CH + CFT 400 + 2	CH + CFT 400 + 4	CH + CFT 400 + 8
0	6.80 ± 0.17	6.80 ± 0.17	6.80 ± 0.17	6.80 ± 0.17	6.80 ± 0.17	6.80 ± 0.17	6.80 ± 0.17	6.80 ± 0.17
24	7.44 ± 0.15 ^a^	7.45 ± 0.15 ^a^	6.63 ± 0.32 ^a^	6.14 ± 0.18 ^a^	2.17 ± 0.81 ^b^	2.48 ± 0.78 ^b^	1.77 ± 0.63 ^b^	1.96 ± 0.59 ^b^
RF ^6^	--	--	0.81	1.3	5.27	4.96	5.67	5.48

Different letters indicate significant differences according to Tukey’s test (*p* ≥ 0.05). An analysis of variance (*p* ≥ 0.05) was performed for each bacterium. ^1^ Treatments: µg/mL. ^2^ Control: *S. aureus* ATCC 27543 without treatment. ^3^ Control: *S. aureus* AMC-43 without treatment. ^4^ Control: *S. aureus* AMC-48 without treatment. ^5^ Vehicle: *S. aureus* ATCC, AMC-43, and AMC-48 containing acetic acid 1%, pH 5.6. ^6^ RF: Reduction factor.

**Table 4:** Biofilm production by bovine-mastitis-associated *S. aureus* and change in bacterial classification due to the antibiofilm effect of N + CFT and CH + CFT combinations.

*S. aureus*	Biofilm Production ^a^	Classification ^b^	OD and Classification After N + CFT Exposition	OD and Classification After CH + CFT Exposition
ATCC 27543	0.29 ± 0.05	(++)	0.06 ± 0.001 (+)	0.11 ± 0.008 (+)
AMC-43	0.25 ± 0.04	(++)	0.05 ± 0.001 (0)	0.18 ± 0.042 (+)
AMC-48	1.28 ± 0.09	(+++)	0.06 ± 0.012 (+)	0.34 ± 0.003 (++)

^a^ Biofilm quantification was determined by OD (595 nm): the average of three replicates obtained from three independent experiments ± standard deviation. ^b^ (0): no biofilm producer; (+): weak biofilm producer; (++): moderate biofilm producer; (+++): strong biofilm producer Stepanović et al. [20].

## Data Availability

The data underlying this article will be shared on reasonable request to the corresponding author.
